# Determinants of COVID-19 Disease Severity–Lessons from Primary and Secondary Immune Disorders including Cancer

**DOI:** 10.3390/ijms24108746

**Published:** 2023-05-14

**Authors:** Antonio G. Solimando, Max Bittrich, Endrit Shahini, Federica Albanese, Georg Fritz, Markus Krebs

**Affiliations:** 1 Guido Baccelli Unit of Internal Medicine, Department of Precision and Regenerative Medicine and Ionian Area—(DiMePRe-J), Aldo Moro Bari University, 70100 Bari, Italy; 2 Department of Internal Medicine II, University Hospital Würzburg, 97080 Würzburg, Germany; 3 Gastroenterology Unit, National Institute of Gastroenterology S. De Bellis, IRCCS Research Hospital, Via Turi 27, 70013 Castellana Grotte, Italy; 4 Department of Anesthesiology, Intensive Care Medicine and Pain Therapy at the Immanuel Klinikum Bernau, Heart Center Brandenburg, 16321 Bernau, Germany; 5 Comprehensive Cancer Center Mainfranken, University Hospital Würzburg, 97080 Würzburg, Germany; 6 Department of Urology and Paediatric Urology, University Hospital Würzburg, 97080 Würzburg, Germany

**Keywords:** COVID-19, SARS-CoV-2, disorder of immunity, cancer

## Abstract

At the beginning of the COVID-19 pandemic, patients with primary and secondary immune disorders—including patients suffering from cancer—were generally regarded as a high-risk population in terms of COVID-19 disease severity and mortality. By now, scientific evidence indicates that there is substantial heterogeneity regarding the vulnerability towards COVID-19 in patients with immune disorders. In this review, we aimed to summarize the current knowledge about the effect of coexistent immune disorders on COVID-19 disease severity and vaccination response. In this context, we also regarded cancer as a secondary immune disorder. While patients with hematological malignancies displayed lower seroconversion rates after vaccination in some studies, a majority of cancer patients’ risk factors for severe COVID-19 disease were either inherent (such as metastatic or progressive disease) or comparable to the general population (age, male gender and comorbidities such as kidney or liver disease). A deeper understanding is needed to better define patient subgroups at a higher risk for severe COVID-19 disease courses. At the same time, immune disorders as functional disease models offer further insights into the role of specific immune cells and cytokines when orchestrating the immune response towards SARS-CoV-2 infection. Longitudinal serological studies are urgently needed to determine the extent and the duration of SARS-CoV-2 immunity in the general population, as well as immune-compromised and oncological patients.

## 1. Introduction

COVID-19 infection is a complex and heterogeneous disease, with the host response crucially determining its course: during the early phase of an infection, there is a substantial production of type 1 interferons, generated at the tissue level by infected as well as surrounding cells [1]. At the same time, SARS-CoV-2 infection can block type 1 interferon signaling in macrophages and dendritic cells [2,3]. Moreover, decreased interferon production—caused for instance by defects in Toll-like receptors (TLRs)—was linked to critically ill COVID-19 patients. Similar clinical courses were observed in patients with alterations in interferon receptors—leading to defective interferon sensing [4,5]. In line with this disease model, Bastard et al. discovered autoantibodies against type 1 interferon, which again hindered sufficient antiviral cellular signaling, in patients suffering from severe COVID-19 disease [4,6]. In general, these traits point towards a crucial role of interferon in preventing severe COVID-10 disease [7]. 

The cytokine production is usually maintained in the first week, when the virus replicates and the most overt clinical symptoms usually appear; next, the antibody production phase takes place together with the expansion of the T-cell compartment. During a viral infection, the peak in T-cell expansion usually occurs about a week from the infection. Consequently, inflammatory mediators are key drivers of COVID-19-related morbidity [1,8]. The knowledge that the cytokines’ role is chiefly driving organ damage was what largely prompted the drug targeting. Commonly, the peripheral blood levels of cytokines are considered trustworthy in reflecting the immune response. Nonetheless, Daamen et al. provided evidence to warrant caution: chemokines and cytokines in peripheral blood differed significantly from those obtained from autoptic lung tissues and bronchoalveolar lavage [9]. This is unlikely to be limited to SARS-CoV-2 and will prompt additional research to map personalized and site-specific patients’ immunomes in infections [10,11]. In this frame of mind, an unbiased immunophenotyping analysis revealed a selective clustering of individuals with severe COVID-19 courses [12]. 

It is possible to draw some distinctions based on the severity of the disease. T-cell activation, DR expression and monocytes are critical; significant differences were found in T-cell activation and MHC DR expression, as well as in the effector memory T-cell population (effector memory T cells re-expresses CD45RA, dubbed TEMRA) [12]. Many studies have investigated the role of immunity in COVID-19 [13,14,15,16,17]. Seminal findings revealed that monocytes correlate with symptoms, with CD169+-activated monocytes lacking in healthy controls [18]. The researchers also discovered a gamma interferon signature (high expression in patients with severe COVID-19 courses), that successfully distinguished patients based on their prognosis.

Furthermore, conditions associated with poor outcomes in COVID-19 appear to be associated with general risk factors, such as type 2 diabetes, obesity and COPD; it is worth noting that these are traditional markers of poor outcomes in almost any severe disease. Aside from organ transplantation, nothing has been discovered to identify the immune system’s state as a major determinant [19]. Chronic inflammation is associated with kidney disease, diabetes and obesity, but these conditions do not result in immunosuppression [20]. Nonetheless, age is the single greatest risk factor for mortality and hospitalization, and immunosenescence represents an intriguing scenario for future research. 

**Figure 1:**
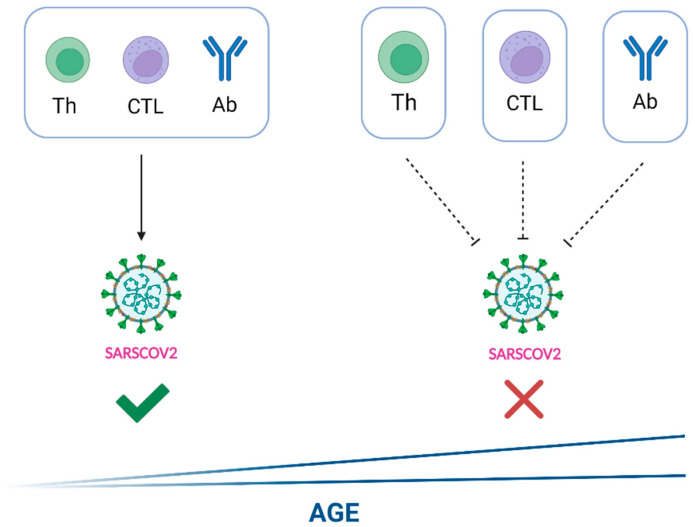
Higher age and a poor adaptive and humoral immune response as determinants of COVID-19 disease severity. Young patients and individuals with sufficient T-cell-based and humoral immune response (left side) usually have self-limiting disease courses. In contrast, older patients and individuals with a limited immune response (right side) have a significantly worse prognosis in terms of COVID-19 outcome. Th: T helper cell; CTL: Cytotoxic T lymphocyte; Ab: Antibody.

Because ACE2 and TMPRSS2 variants and expression can be candidates for gender and country differences in COVID-19 severity, host genetics is also important in COVID-19 [20,21]. There are two susceptibility loci for severe COVID-19 with respiratory failure [21]. Nonetheless, we review the current evidence pointing to novel aspects of immune-related conditions that may influence the outcome of SARS-CoV-2 infections (Figure 1).

## 2. Immunity, Immune Aging and COVID-19

### 2.1. Immune Response against SARS-CoV-2

Although infection with SARS-CoV-2 was expected to activate the host’s immune response, data on this specific trait were initially scarce [22,23]. While SARS-CoV-2 infection results in both humoral and cellular immunity [24], the T-cell response does not differ significantly between mild and severe forms. Cross-reactions have been reported in unexposed subjects, most likely due to non-SARS-CoV-2 coronaviruses [24]. Compared to seronegative counterparts, seropositive individuals are significantly more protected against infection. Thus, the strong sequence homology and structural similarity with SARS-CoV-1 initially supported genetic and structural modelling, revealing an epitope-level similarity prediction. Despite being slightly less effective than mRNA vaccines (95%) [25,26,27], the humoral defense is protective against the virus in much the same way as the adenovirus-vectored vaccine (70%). 

A test designed to determine which pieces of SARS-CoV-2 are recognized by the immune system was supported by CD4+ and CD8+ epitopes predicted to play significant roles in SARS-CoV-2 [22]. Specific analysis of SARS-CoV-2 human CD4 and CD8 T-cell epitope data proved 1400 additional SARS-CoV-2 epitopes and suggested different immunodominant regions of the virus as well as more commonly recognized epitopes. 

T-cell and antibody responses in COVID-19 cases appear to be orchestrated by two main principles: in most humans, SARS-CoV-2 causes acute infections that resolve or cure. Both antibodies and T cells are important in modulating humoral responses, human vaccines and protecting against the virus [26]. A better understanding of SARS-CoV-2 T-cell and antibody adaptive responses prompted further investigation, leading to the measurement of SARS-CoV-2 immunity while identifying epitope pools detecting CD4+ in 100% and CD8+ T cells in 70% of convalescent COVID-19 patients. Furthermore, T-cell responses were discovered to be focused not only on the spike protein but also on M, N and other ORFs [28,29]. Additional details on the impact of different viral strains on the described groups would be informative in understanding disease severity and patient outcomes. For instance, recent studies have highlighted the importance of considering the viral strain in the context of COVID-19 prognosis. One study found that patients infected with the B.1.1.7 (Alpha) variant had a higher risk of hospitalization and death compared to patients infected with the original strain. Another study found that the B.1.351 (Beta) variant was associated with an increased risk of reinfection in patients who had previously recovered from COVID-19. Therefore, further research on the impact of different viral strains on disease severity and patient outcomes is needed to develop effective treatment strategies [30,31]. 

Of note, T-cell reactivity to SARS-CoV-2 epitopes has also been observed in non-exposed individuals [23]. In acute COVID-19 cases, disease severity correlates with adaptive immunity to SARS-CoV-2 underlining that a coordinated immune response is protective against the virus. T cells appear to be the major contributors to controlling SARS-CoV-2 infection. As the first line of defense against pathogens, the innate immune system plays a crucial role in combatting this novel virus. To better understand the interaction between SARS-CoV-2 and the human innate immune system, a conceptual framework is needed to link clinical observations with experimental findings from the first year of the pandemic. It has been observed that variations in innate immune system components among individuals contribute significantly to the diverse disease courses seen in COVID-19. Therefore, understanding the pathophysiological mechanisms of the cells and soluble mediators involved in innate immunity is essential to develop effective diagnostic markers and therapeutic strategies for COVID-19. However, more research is needed to establish the causality of events, which is currently lacking [32]. Several pathophysiological mechanisms of the innate immune system are involved in COVID-19. One of the most prominent mechanisms is the overactivation of the immune response, leading to a cytokine storm, which can cause severe damage to the lungs and other organs. This overactivation of the immune response can also result in the infiltration of immune cells, such as macrophages and neutrophils, into the lungs, leading to inflammation and tissue damage. Moreover, it has been observed that the innate immune system detects viral RNA through pattern recognition receptors, such as Toll-like receptors (TLRs) and retinoic acid-inducible gene 1 (RIG-I)-like receptors (RLRs), leading to the production of type I interferons (IFNs). However, SARS-CoV-2 can evade the innate immune system by inhibiting the production of type I IFNs, leading to uncontrolled viral replication and dissemination. In addition, there is evidence to suggest that the complement system, which is part of the innate immune system, is activated in COVID-19. Activation of the complement system can lead to the recruitment of immune cells, including neutrophils and macrophages, to the site of infection, contributing to inflammation and tissue damage. Furthermore, recent studies have suggested that the innate immune system may also play a role in the long-term effects of COVID-19, such as persistent fatigue and cognitive impairment. It has been proposed that the innate immune system may contribute to these long-term effects by inducing chronic inflammation and oxidative stress. Overall, a better understanding of the pathophysiological mechanisms involved in the innate immune system’s response to SARS-CoV-2 is essential to develop effective diagnostic markers and therapeutic strategies for COVID-19 [33,33,34].

The adaptive immune system has three branches in its arsenal against the virus, but there is no strong evidence of a causal negative association between adaptive immunity and disease severity. Conversely, age is a major COVID-19 risk factor, and adaptive immunity shortcomings seem to be part of the dysfunctional response when poorly coordinated T-cell responses and limited naïve T cells are in place [35].

When considering the immune system ageing, we primarily consider T cells and thymic involution. The naive T-cell compartment contracts, thymus cells acquire senescent characteristics and some expanded oligoclonal T-cell populations are exhausted [36]. These phenomena also include herpes virus infections and susceptibility to bacteria [37]. The myeloid compartment also ages, as Lissner et al. demonstrated ex vivo and in vitro [38]. The authors used the Listeria monocytogenes infection as a model to assess the impact of a patient’s age on the course of the disease.

Listeria infection is usually insignificant in most young adults. However, after the age of 80, the risk of stroke and poor outcome increases significantly [38,39]: in vitro, baseline differences in the monocyte response to listeria were significant between older and younger adults. Without any prior stimulation, older adults were already overproducing inflammatory cytokines. This specific trait has also been confirmed in bulk and single-cell analysis of PTX3 expression in COVID-19 patients’ peripheral blood and lung tissue [40]. Regarding the six-hour time point, older adults had a longer persistence of inflammation in their monocytes. This finding sparked several investigations, including COVID-19 studies, into the myeloid compartment [37]. Corticosteroids are thus the foundation of therapeutic strategies to reduce mortality in clinical settings of COVID-19 respiratory failure. The discovery that age can influence immunological fitness prompted further research in a broader field typically associated with immunodeficiency and immune dysregulation. 

T cells have been shown to contribute to a better outcome in SARS-CoV-2 infections with lower viral loads [41] and play an important role in the immune response [37]. In the monkey model, CD8+ T cells provide control. Individuals with agammaglobulinemia and B cell deficiency have a moderately increased risk of hospitalization. Moreover, COVID-19 is mostly mild in people with multiple sclerosis who take ocrelizumab (an antiCD20 antibody). In contrast, in the absence of detectable neutralizing antibodies, one single dose of Moderna or Pfizer vaccine provided substantial protection in most individuals [42]. Following that, researchers hope to determine how long SARS-CoV-2 immunological memory lasts. Dan et al. conducted an extensive analysis, highlighting significant immune memory in most individuals 8 months post-COVID-19 infection. Memory kinetics differ between T cells, memory B cells and antibodies. Immune memory is complex and heterogeneous, with an estimated half-life with a wide confidence interval due to COVID-19 heterogeneity and approximately 5% of individuals having low-level immune memory at 6 months [43].

### 2.2. Lessons from HIV Infection

The T-cell count has been discovered to be important in viral load control and infection eradication [44]. However, the first study on COVID-19-infected HIV patients from Spain found no significant difference between mild, moderate and severe courses due to HIV-related T-cell count. In large HIV cohorts, there were no differences in hospitalization rates, either. There was no dependence on T-cell count, specifically CD4+ T-cell count: Ho et al. reported a slightly higher rate of inflammatory cytokines but no differences in hospitalization in HIV-positive versus HIV-negative COVID-19 patients, and they were unable to identify a T-cell count that conferred an increased risk [45]. HIV infection, on the other hand, has been linked to a twofold increase in the risk of death in COVID-19-infected individuals in another study [46]. As a result, it is attractive to speculate that there is a disease spectrum associated with HIV viral load, immunosuppression, hospitalized cases and tuberculosis exposure that could explain clinical heterogeneity. Finally, HIV does not appear to be a major risk factor for highly aggressive COVID-19 courses, establishing a pathobiological stage in which T cells do not lead to disease aggressiveness. However, the frailty of this population subgroup prompted further research into vaccination and its prioritization for vulnerable patient subgroups. 

Many studies have investigated the impact of HIV on COVID-19 outcomes and have suggested that the close monitoring of immune status and viral load, as well as adjustments to HIV treatment regimens, may be necessary for optimal management of COVID-19 in HIV-positive individuals. Additionally, early and aggressive treatment with antiviral therapy and corticosteroids has improved outcomes in COVID-19 patients, including those with HIV. COVID-19 vaccines are also recommended for HIV-positive individuals, although more research is needed to fully understand their safety and efficacy in this population. Finally, psychological and social support services are important considerations for HIV-positive individuals with COVID-19, as they may face unique challenges related to social isolation and stigma [47].

### 2.3. Vaccination and Immunity

The underlying trained innate immunity represents a significant breakthrough in COVID-19 prevention [48,49]. SARS-CoV-2 infection was tracked prospectively in 3720 healthcare workers who received two doses of the BNT162b2 vaccine between January 18 and March 31, 2021, with data collected until May 10, 2021. In subjects with symptoms suggestive of SARS-CoV-2 infection or contact with an infected subject, nasopharyngeal swabs were collected and tested for SARS-CoV-2 RNA positivity [25]. Surprisingly, subjects who had previously been infected and then vaccinated, thus receiving triple exposure to viral antigens, could benefit from a 50% risk reduction. All SARS-CoV-2-exposed subjects had a sustained level of SARS-CoV-2 neutralizing antibodies against all variants tested [25,50]. Significant responses to 25 mcg of Moderna mRNA-1273 vaccination spike were observed, as were RBD IgG antibody responses lasting up to 7 months [51]. A significant response has also been reported at various time points, higher at day 43 compared to day 15 and lasting up to day 209 [51]. 

CD4+ and CD8+ T cells exhibited similar behavior. The quality and duration of vaccination responses with various vaccine platforms have also been studied. Moderna, Pfizer, Johnson & Johnson, AstraZeneca and Novavax have all been tested for their ability to establish and maintain immune responses over time [52,53]. This will be useful in determining the magnitude and duration of vaccine-induced immunity, as well as in comparing different vaccine platforms in terms of responses induced by each other and in comparison to natural infection. Furthermore, this will help future vaccine designers determine when and if a booster is needed, as well as develop dose-sparing strategies [54]. Tarke et al. identified 280 different CD4+ restricted epitopes in this frame of mind (dominant epitopes are highly promiscuous, with implications for population coverage). They also discovered 523 distinct CD8+ epitopes, each recognizing multiple epitopes and antigens, yielding a conservative estimate of 15–20 epitopes recognized per donor, with important implications for viral immune escape [55].

## 3. Immune Dysregulation and Severity of COVID-19 Infection

### 3.1. COVID-19 in Patients with Cancer—Clinical Data, Risk Factors and Vaccination Response

When researchers examined factors contributing to COVID-19 mortality in European cancer patients, they discovered that onco-hematological pathologies, metastasis and additional comorbidities were risk factors for developing the severe disease [56]. Nonetheless, the retrospective nature of the available data significantly affected its quality. Furthermore, survival from symptom onset in hospitals was not influenced by a cancer diagnosis per se but rather by suboptimal ICU measures used on cancer subjects versus non-oncologists used on COVID-19 patients also suffering from cancer compared to COVID-19 patients without cancer [57]. Table 1 summarizes the available evidence on this topic.

Kuderer et al. defined factors associated with COVID-19 mortality in cancer patients, describing a not significant increase in risk for non-cytotoxic therapy (targeted agents, endocrine therapy, immunotherapy, radiation). At the same time, they confirmed increased mortality for subjects with hemato-oncological conditions, the elderly, and patients with comorbid conditions [61,65]. Large studies were initiated to examine iatrogenic immunosuppression, which surprisingly excluded an additional risk conferred by active chemotherapy for COVID-19 hospitalization severity or mortality. Indeed, a larger study by Kuderer et al. identified progressive disease as a risk factor for COVID-related mortality [61]. The authors’ findings are consistent with the CDC’s risk stratification for mortality [19]. Nonetheless, they discovered that, besides already known secondary effects [66], immune checkpoint inhibitor-based therapies are linked to an increased risk of mortality and disease severity. Remarkably, chemotherapy did not affect prognosis in this study [61]. 

Regarding humoral and cellular immunity, cancer patients are no exception to the general concept of vaccine responsiveness [67]. Passive immunization is also an option for subjects with ineffective vaccine responses [68]. In COVID-19 cancer patients, older age, the number of comorbidities, ECOG PS ≥2, active cancer and chemotherapy alone or in combination all increase the risk of death. 

There is very little information available on the antibody responses against SARS-CoV-2 in cancer patients. The first prospective multicenter observational study evaluated the antibody response in cancer patients and oncology healthcare workers with confirmed or clinically suspected COVID-19 [69], demonstrating that cancer patients infected with SARS-CoV-2 have IgG antibody responses comparable to subjects not suffering from cancer. SARS-CoV-2 laboratory testing that is both timely and accurate is critical in managing COVID-19. Combining antibody testing and RT-PCR on swab specimens may improve COVID-19 detection [69]. This evidence was corroborated by Kamar et al., who discovered that three doses of an mRNA COVID-19 vaccine were effective in organ transplant recipients [70]. 

A prospective, multicenter cohort study conducted in 2022 aimed to compare the spike IgG seropositivity rate in blood samples from 776 cancer patients and 715 non-cancer volunteers following inactive SARS-CoV-2 vaccination. The cancer patient group had a seropositivity rate of 85.2%, while the control group had a rate of 97.5%. Cancer patients not only had a significantly lower seropositivity rate but also lower antibody levels (*p* < 0.001). Finally, lower seropositivity in cancer patients was associated with age and chemotherapy (*p* < 0.001) [71].

A study from 2021 aimed to assess the SARS-CoV2 IgG seroprevalence in 74 older patients (aged ≥ 80 years) with cancer one month after receiving the second dose of the BNT162b2 vaccine. While median serum IgG levels in older cancer patients were lower compared to control (2396.10 AU/mL vs. 8737.49 AU/mL; *p* < 0.0001), this study still was the first to describe a positive immune response in this vulnerable patient subgroup [72]. A 2022 meta-analysis, on the other hand, looked at factors predicting poor seroconversion in 5499 cancer patients. The authors discovered that age, male gender and metastatic disease were associated with a lower seropositivity after COVID-19 vaccination. Additionally associated with seropositivity were immunoglobulin heavy chain variable mutation status and high concentrations of IgG, IgM and IgA. Regarding cancer treatment strategies, anti-CD20 therapy within the last 12 months and chemotherapy were found to be negatively associated with seroconversion. These findings suggested that improved vaccination strategies would be beneficial for the elderly, males or patients receiving active chemotherapy and that prevention should be prioritized even after a full course of vaccination [73]. Another recent study examined COVID-19 vaccine uptake trends in 579 sequential cancer patients previously infected with SARS-CoV-2. Specifically, older age and female sex were significantly associated with higher vaccine uptake in univariate and multivariate models (age (OR = 1.18, *p* < 0.001), and female sex (OR = 1.80, *p* = 0.003), respectively) [74]. 

Notably, the aim of Mai et al.‘s systematic review and meta-analysis was to determine the proportion of non-responders to COVID-19 primary vaccination in 849 patients with hematological cancer and 82 patients with solid cancer who seroconverted after a booster dose. Seroconversion occurred in 44% of patients with hematological malignancies, while a significantly higher seroconversion (80%) was observed among solid tumor patients. Higher antibody titers were found to be significantly associated with an increased duration between the second and third dose (OR = 1.02, *p* ≤ 0.05), patient age (OR = 0.960, *p* ≤ 0.05) and cancer type. Therefore, administering a COVID-19 vaccine booster dose improved seroconversion and antibody levels. Patients with solid cancer consistently responded better to booster vaccines than patients with hematological cancer [75]. 

Another recent systematic review and meta-analysis of 28 articles assessed the efficacy and safety of COVID-19 vaccines in patients with active malignancies. In contrast to the previous meta-analysis, they discovered higher overall seroconversion rates of 70% and 88% in patients with solid tumors and hematologic malignancies, respectively, after receiving a second dose of the COVID-19 vaccine [76].

In particular, a recent study compared the development of neutralizing antibodies against SARS-CoV-2 in non-vaccinated patients with multiple myeloma (MM) and COVID-19 to patients who received two doses of the BNT162b2 vaccine, most likely due to immunoparesis [77,78]. Patients with MM and COVID-19 had a better humoral response than vaccinated patients with MM. COVID-19-positive patients had a higher median neutralizing antibodies titer than vaccinated patients (87.6% vs. 58.7%; *p* = 0.01). However, there was no difference in neutralizing antibody production between COVID-19-positive and vaccinated patients who did not receive treatment (*p* = 0.14). As a result, it was suggested by the authors that vaccinated patients with MM on treatment who have not previously received a COVID-19 infection should be considered for booster vaccination [79]. 

There is little information in the literature about the levels of autoantibodies in patients with paraneoplastic syndromes and whether these autoantibodies could interfere with the vaccine response in any fashion.

Immunologic self-tolerance defects increase the risk of paraneoplastic autoimmune diseases and immune-mediated toxicity, which have been examined in patients with thymic epithelial tumors. Common COVID-19 vaccine adverse events among 54 participants in a 2021 US study included injection site pain, fatigue and headaches [80]. Among the 19 patients previously been diagnosed with paraneoplastic autoimmune disease, 3 experienced autoimmune flares after the first dose, and 3 experienced autoimmune flares after the second dose. The majority of paraneoplastic autoimmune disease flares were mild and self-limiting. Following vaccination, one patient (2%) was diagnosed with a new paraneoplastic autoimmune disease. As a result, the overall tolerability of COVID-19 mRNA vaccines in patients with thymic tumors was comparable to that of the general population [78,79]. 

Prioritizing these populations who will benefit the most from SARS-CoV-2 vaccination and thus have a positive impact on the pandemic’s trajectory is critical. Patients with cancer, immunological disorders and close contacts should be prioritized. Customizing vaccination schedules could be one approach to developing more effective health policies based on evidence-based data. Alternative vaccine schedules are not insignificant, and they frequently affect vaccine efficacy. Similarly, adjusting the schedules to account for the risk of severe COVID-19 outcomes, as well as individuals’ ability to mount and sustain an immune response, is critical [81,82].

### 3.2. Immune Dysregulation and Severity of COVID-19 Infection 

Researchers also investigated the potential contribution of systemic autoimmune disease (SAD) to COVID-19 disease severity. For this, they investigated the prognostic impact of SAD on COVID-19 mortality in a nationwide Spanish registry study performed before the introduction of SARS-CoV-2 vaccination. The presence of SAD in COVID-19 patients was generally associated with higher mortality. However, after adjusting for patient characteristics and comorbidities, SAD did not have a statistically significant effect on mortality [83]. While case-control studies did not confirm an isolated effect of autoimmune disease on COVID-19 severity, further population-based studies implied higher mortality in SAD patients—with advanced age, male gender and comorbidities again being the relevant predictive factors [84]. 

The use of biologics and other immunosuppressive therapies has been a concern during the COVID-19 pandemic, as it was initially believed that these treatments could increase the risk of severe disease. However, studies have shown that the risk factors for COVID-19 hospitalization are similar in patients receiving biologics compared to the general population [85]. Treatment with biologics has not been found to affect the severity of COVID-19 [6,86]. In some cases, patients with COVID-19 were found to have autoantibodies to type I interferons, which can contribute to uncontrolled inflammation and worsen disease severity [1,5,6,16]. In patients with autoimmune diseases, the use of biologics and other immunosuppressive therapies is common. However, it is important to balance the need for disease control with the potential risk of infection. A study by Haberman et al. found that treatment with TNF inhibitors, IL-17 inhibitors, IL-23 inhibitors, IL-12 inhibitors and JAK inhibitors did not increase the risk of COVID-19 severity [87]. These cytokine inhibitors may even be beneficial in preventing endothelial toxicities and systemic complications associated with COVID-19 [87,88]. While iatrogenic immunosuppression can increase the risk of infection, it is also important to consider the potential role of autoimmunity and uncontrolled inflammation in COVID-19 severity. Inflammation and immunity play important roles in medicine, including the systemic inflammatory chronic state [89]. Therefore, interception of autoimmunity and uncontrolled inflammation may be a potential therapeutic strategy for severe COVID-19 cases [5,90,91].

Recent studies have also shown that the IL-31/IL-33 axis plays an essential role in the immune response against SARS-CoV-2. In particular, the axis is involved in the regulation of cytokine production and immune cell activation [92]. Therefore, further research is needed to investigate the potential therapeutic targets for COVID-19 using IL-31/IL-33 axis modulation. Understanding the immune response to SARS-CoV-2 in different patient populations, including cancer patients and those with immune deficiencies, is critical in developing effective treatment strategies. The impact of different viral strains on disease severity and patient outcomes should also be considered. The IL-31/IL-33 axis has emerged as a key player in the immune response against SARS-CoV-2, and further research is needed to fully understand its potential as a therapeutic target for COVID-19 [93]. In summary, while the use of biologics and other immunosuppressive therapies was a concern during the COVID-19 pandemic, studies have shown that these treatments do not increase the risk of COVID-19 severity. Additionally, in patients with autoimmune diseases, cytokine inhibitors may even be beneficial in preventing complications associated with COVID-19. The potential role of autoimmunity and uncontrolled inflammation in COVID-19 severity highlights the importance of considering these factors in therapeutic strategies. More research is needed to better understand the complex interaction between SAD and COVID-19—this is also reflected by the recent discovery of SAD—and cancer-specific antinuclear antibodies in COVID-19 patients [94]. Moreover, Böröcz et al. recently reported compound-dependent expression levels of natural autoantibodies after COVID-19 vaccination [95]. 

The discovery of inborn errors in type I IFN immunity in patients with life-threatening COVID-19 paved the way for further research in this area [5]. Inborn errors of immunity are an archetypical field that should be further investigated in terms of patient care and a better understanding of the host defense against SARS-CoV-2. Despite emphasizing the lack of high-quality evidence, the CDC lists primary immune deficiencies as a risk factor [19]. Immune deficiencies are listed as comorbid conditions in three large studies, but they are not further defined, and there is a lack of pre-specified iatrogenic immune-compromising conditions [96]. Focusing on B cells, Quinti et al. began with a small cohort of seven patients with antibody deficiency—two patients had agammaglobulinemia with no B cells, and five patients suffered from common variable immunodeficiency (CVID): one of seven patients died [97,98]. The two patients with agammaglobulinemia, in the authors’ opinion, had a better outcome, including a shorter hospital stay, than the patients with CVID [97]. In line with this, Soresina et al. described two individuals with X-linked agammaglobulinemia, another condition characterized by the absence of B cells; both subjects developed pneumonia which was presumed to be bacterial but recovered uneventfully and did not seem to have any evidence of ARDS or any evidence of complications leading to ICU admission [98]. 

Based on this evidence, it is tempting to conclude that B cells can be harmful, especially considering additional evidence regarding acalabrutinib intake. Even though the study was small and uncontrolled, the authors assumed that acalabrutinib was extremely beneficial in their COVID-19 cohort [99]. Moreover, the authors proposed that the underlying mechanism could be IL-6 monocytic production, with IL-6 levels decreasing in treated patients [99], as confirmed by in silico analyses [1,100]. Nevertheless, achieving the statistical power required to corroborate conclusions about inborn errors of immunity is difficult.

Meyts et al. were the first to describe 94 patients with primary immunodeficiency (PID, IEI) and coinfection with COVID-19. Specifically, COVID-19 was found in 20–25% of IEI patients, with 75–80% developing mild SARS-CoV-2 disease [101]. IEIs, in particular, could predispose patients to more severe forms of COVID-19, with a variable, unpredictable disease course in cases of antibody deficiency. Overall, 60 patients were hospitalized with a mortality rate of 11%, comparable to the general population (between 10% and 13%). When comparing the CDC and the Meyts’ cohort mortality rates, it is important to compare the age of the patients who died, as well as the per cent mortality: age is the prognostically most relevant risk factor. The key points highlight a link between age and clinical phenotype, with young kids being relatively resilient. A two-year-old with a chronic granulomatous disease that was not diagnosed had hemophagocytic lymphohistiocytosis and Burkholderia cepacia infection at the time of death; however, we do not know the precise impact of COVID-19 in this specific case [101]. The second, young combined-immune deficiency patient died from sepsis and hemophagocytic lymphohistiocytosis [101]. The underlying pathobiology was unclear. Ancillary to these examples, two patients with antibody deficiency had cardiomyopathy or lymphoma, and two subjects experienced sepsis, renal failure and heart failure as the ultimate cause of death [101], as published recently [102,103,104]. Moreover, two patients with antibody deficiency, lung disease and heart disease as comorbid conditions died because of sepsis and renal failure, respectively. Despite this heterogeneous cohort, a proportion of 7% HLH, 6% renal insufficiency and 4% autoimmune cytopenias are unquestionably higher than in the general population. This raises the possibility of an increased risk of autoimmune diseases, such as autoimmune cytopenias and Guillain–Barré neuropathy. 

Collectively, we may pave the way for representing two major causes of death, namely HLH in younger subjects and sepsis or renal failure in older subjects [101]. Buccioli et al. confirmed these fundings. Indeed, 57% of SCID pre-HSCT and 75% of Good syndrome subjects were admitted to ICU. Subjects with the autoimmune polyendocrine syndrome type 1 (APS) behaved similarly, with 15% mortality [101]. At baseline, APS (APECED) with autoimmune polyendocrine syndrome type 1, mucocutaneous candidiasis, hypoparathyroidism and hypoadrenocorticism appear to neutralize anti-IFN antibodies (alpha or omega). FACT, rare genetic variants with compromised IFN type I immunity, reduced the production of TLR3-, MDA5- and APECED activity and reduced the cellular response to STAT1- and STAT2-signaling [105]. In these circumstances, it appears that a compromised immune response is the driving force behind more severe forms of COVID-19.

Hypersecretion of IFN-I, on the other hand, causes severe forms of Multisystem Inflammatory Syndrome in Children (MIS-C), a SOCS1-driven condition [106]. Additionally, type I IFN appears to be related to pernio (chilblains) severity in COVID-19 [107].

Other IEIs, such as X-linked agammaglobulinemia or states with reduced or absent B lymphocytes, have a lower ability to eliminate the virus with a longer duration of infection [108]. In these cases, combining monoclonal antibodies and antiviral drugs can ensure a good outcome. They are not more severe in other forms of the disease, such as phagocyte disorders, autoinflammatory diseases and hereditary angioedema.

Furthermore, data from the IPINet network’s 2021 and 2022 studies on a large population of Italian subjects with IEI revealed 74 cases of SARS-CoV-2 infection in 1161 patients with CVID, with an incidence comparable to that of the general adult population. The cumulative incidence is even lower in the pediatric population [109,110]. In terms of the severity of the infection in patients with IEI (IPINet, 2021), according to age, subjects <18 years have a lower incidence of severe symptoms than older subjects, with no deaths following the infection in patients <30 years. 

The mortality rate was higher in Good syndrome and Del 22q11, both associated with a T-cell defect [109,110]. Four deaths were reported in the adult age group in the IPINet, with an overall death rate from COVID-19 of 3.5% in IEI vs. 2.5% in the Italian population. The median age at death in subjects >18 years with IEI was 48 years vs. 80 years in the Italian population, with a range from 5.7% in CVID to 33% in Good syndrome. Mortality was also higher in the 50–60-year age group (14.3 vs. 0.6%). Greater comorbidities at a younger age appear to best distinguish these patients from the general population [109,110]. STAT1 alterations are no exception, potentially posing a life-threatening situation, resulting in bone marrow failure and poor outcomes [91,111]. This bone marrow failure phenotype [90,112,113,114] seems particularly relevant due to the possibility of targeting the JAK/STAT pathway in COVID-19 [115].

The risk factors for increased severity of COVID-19 suggest that a more severe outcome correlates with the same risk factors seen in the general population, namely the male sex and associated comorbidities, such as chronic lung disease and chronic liver disease. On the contrary, rheumatological manifestations would not affect mortality. The same factors determine the duration of the infection (2 weeks vs. 2–3 months) [116]. This may be a risk factor for viral spread.

## 4. Conclusions

The immunocompromised patient is an excellent candidate for an intense clinical investigation into the relationship between SARS-CoV-2 infection and host defenses. Three emerging paradigms in cancer treatment prioritization and frailty have a greater impact on the clinical outcome than cancer-related immune dysfunction. Of note, cancer-related immune dysfunction did not play a crucial role in COVID-19 disease severity in contrast to cancer-related frailty. SARS-CoV-2 infection was contracted by 20–25% of subjects with inborn errors of immunity, with 75–80% having a mild or asymptomatic clinical course. COVID-19 severity and mortality appear to be associated with comorbidities that manifest younger than the general population.

Ultimately, the COVID-19 vaccines are safe for cancer patients, but they may be less effective than in healthy people, especially those with compromised immune systems.

## Figures and Tables

**Table 1:** Available data obtained from SARS-CoV-2 infection and cancer.

Study	N, Neoplasms	Type	Risk Factors of Severe Outcome
China, Hubei [56]	205, mixed	Retrospective	Hematological tumors, chemo <4 weeks, metastatic disease progression
China, Wuhan [57]	232, mixed	Case-matched control	Advanced stage, ECOG, older patients
China, Wuhan [58]	28, mixed	Retrospective	Antineoplastic tx <2 weeks, CT patchy consolidation
France, Paris [59]	76, breast cancer	Prospective registry	Hypertension, older age
France, Lyon [60]	302, mixed	Retrospective	Male gender, ECOG, PD cancer
CCC19 (USA, Canada, Spain) [61]	928, mixed	Crowdsourcing	Age, male gender, NCDs, ECOG, PD cancer, smoking
TERAVOLT [62]	200, thoracic cancer	Crowdsourcing	Smoking
US, NYC [63]	20, childhood cancer	Retrospective	No increased risk of infection vs. non-cancer patients
US, NYC [64]	334, mixed	Case-matched control	Older age

## Data Availability

Not applicable.
